# A New Chaotic System with a Self-Excited Attractor: Entropy Measurement, Signal Encryption, and Parameter Estimation

**DOI:** 10.3390/e20020086

**Published:** 2018-01-27

**Authors:** Guanghui Xu, Yasser Shekofteh, Akif Akgül, Chunbiao Li, Shirin Panahi

**Affiliations:** 1 School of Electrical and Electronic Engineering, Hubei University of Technology, Wuhan 430068, China; 2 Hubei Collaborative Innovation Center for High-efficiency Utilization of Solar Energy, Hubei University of Technology, Wuhan 430068, China; 3 Faculty of Computer Science and Engineering, Shahid Beheshti University, Tehran 1983969411, Iran; 4 Department of Electrical and Electronic Engineering, Faculty of Technology, Sakarya University, Serdivan 54187, Turkey; 5 Jiangsu Key Laboratory of Meteorological Observation and Information Processing, Nanjing University of Information Science & Technology, Nanjing 210044, China; 6 School of Electronic & Information Engineering, Nanjing University of Information Science & Technology, Nanjing 210044, China; 7 Department of Biomedical Engineering, Amirkabir University of Technology, Tehran 1591634311, Iran

**Keywords:** chaotic systems, circuit design, parameter estimation, optimization methods, Gaussian mixture model

## Abstract

In this paper, we introduce a new chaotic system that is used for an engineering application of the signal encryption. It has some interesting features, and its successful implementation and manufacturing were performed via a real circuit as a random number generator. In addition, we provide a parameter estimation method to extract chaotic model parameters from the real data of the chaotic circuit. The parameter estimation method is based on the attractor distribution modeling in the state space, which is compatible with the chaotic system characteristics. Here, a Gaussian mixture model (GMM) is used as a main part of cost function computations in the parameter estimation method. To optimize the cost function, we also apply two recent efficient optimization methods: WOA (Whale Optimization Algorithm), and MVO (Multi-Verse Optimizer) algorithms. The results show the success of the parameter estimation procedure.

## 1. Introduction

A chaotic system has been considered with great potential in engineering applications, in which many chaotic systems with different properties have been studied. Specifically, some systems have the properties of amplitude control and offset boosting [1,2,3,4]. In this paper, we use a new three-dimensional (3D) chaotic system in random number generation and signal encryption, which are important engineering applications of chaotic systems [5,6,7,8,9,10]. To do this, an electronic design of the system is implemented as a real electronic circuit to generate random numbers. Finally, the one-dimensional (1D) and two-dimensional (2D) parameter estimation of the system is reported based on a non-traditional parametric model cost function and two new optimization methods. 

The topic of self-excited and hidden attractors is a new attractive topic in dynamical systems [11,12,13]. Recent studies have classified dynamical attractors as self-excited or hidden [14,15,16,17]. A self-excited attractor has a basin of attraction which intersects with at least one unstable equilibrium. If that is not the case, the attractor is hidden [18,19,20,21]. According to the above definition, most of the classical chaotic attractors are self-excited [22,23]. It has been demonstrated that the attractors in dynamical systems with no equilibria [24,25,26,27,28,29,30,31,32], with stable equilibria [33,34], with lines [35,36] and curves of equilibria [37,38,39,40,41], and with plane [42] or surface of equilibria [43] are hidden attractors. Even some systems can belong to more than one category [3,44,45,46,47]. Hidden attractors cannot simply be located. There have been some efforts in literature to solve this problem [18,48,49].

As we know, modeling of the real world chaotic systems has received great attention in recent decades [50,51,52,53,54,55]. Choosing proper values for model parameters is essential in chaotic systems, since they are very sensitive, both to model parameters and initial conditions. A slight change in the parameters of the chaotic system may cause important bifurcation in its behavior, because of the butterfly effect of the chaotic system [56]. Therefore, the parameter estimation problem of chaotic system models is a complex problem [57,58,59].

There are some widely used methods for the parameter estimation of the chaotic systems which are based on optimization methods [60,61,62]. In these methods, the problem of the parameter estimation is generally formulated as a cost function based on an error function between a time series obtained from a real system and a time series obtained from a known model with unknown parameters of that system. The goal of the parameter estimation method will then be to find the best values of the unknown parameters of the model which minimize the cost function. In addition, the optimization approaches have been used algorithms for this problem to find the best values of the unknown parameters as quickly as possible. They are algorithms such as genetic [63], particle swarm optimization [64], and evolutionary programming [65]. However, approaches that utilize cost function based on the error function seem to bear major limitations because of the butterfly effect of the chaotic systems [57,58,59].

It was remarked that the state space would be a proper domain to analyze the chaotic systems rather than the time-domain. The time series generated by the chaotic systems have random-like behavior in the time-domain, but they are ordered in the state space. They can show specific topologies in the state space named strange attractors. In this paper, we use a non-conventional metric as a useful cost function for the parameter estimation method. Accordingly, we model the attractor distribution of a real chaotic system by a parametric model named the Gaussian mixture model (GMM). It can provide flexible and probabilistic modeling for data distributions. GMM is also a commonly used parametric model in the pattern recognition and machine learning domain [66]. For example, in the speech recognition field, a set of GMMs was introduced to model phone attractors in a reconstructed phase space (RPS) in which the RPS is a time-independent domain similar to the state space [67,68,69]. The phone classification results showed that the GMM could be a useful model to capture the structure and topology of the speech attractors in the RPS. In addition, models of Gaussian mixture were recently used as the parameter identification method for some chaotic systems [70,71,72].

Here, to optimize the cost function, two recent efficient optimization methods are applied, including the WOA (Whale Optimization Algorithm), and MVO (Multi-Verse Optimizer) algorithm. Also, for testing the parameter estimation method in the chaotic systems, a real circuit is utilized based on a new chaotic system in this paper. All the data (time series) are obtained from the circuit that is designed based on the new chaotic system.

The contributions of this paper are described as:

- A new 3D chaotic system with saddle equilibriums is proposed by a set of ordinary differential equations.
- Dynamical properties of the 3D chaotic system are then reported that exhibit its dynamics.
- The electronic circuit implementation of the 3D chaotic system is studied and used to present a random number generator (RNG), and its signal encryption is then introduced as an engineering application.
- 1D and 2D parameter estimation of the electronic circuit is done by a GMM based cost function.
- The cost function is optimized using two new efficient optimization methods called the WOA and the MVO algorithms.
- By comparing the experimental data with numerically generated time series, the best-fitting parameters are found because the circuit had (almost) the same dynamics as the 3D chaotic system.

The structure of the paper is organized as follows: in the next section we introduce and analyze the new chaotic system with saddle equilibriums. In Section 2, we investigate it carefully through bifurcation analysis, spectrum of Lyapunov exponents, and its entropy. Section 3 deals with the circuit implementation of this new system and a real circuit application based on mobile RNG design. In the next section, the cost function based on the GMM is introduced. Two meta-heuristic optimization algorithms (WOA and MVO) are presented in Section 5. Results of the cost function and the parameter estimation of the new chaotic system using the WOA and MVO methods are available in Section 6. Finally, Section 7 is the conclusion of the paper.

## 2. A New Chaotic System and Its Analysis

In this section we introduce a new 3D system which can show chaotic behavior. Consider a system described with the following ordinary differential equations:

(1) $$\begin{array}{l} \dot{x}=gz\\ \dot{y}=dx^{2}+ey^{2}-f\\ \dot{z}=-ax-bx^{2}+cy^{2} \end{array}$$

**Figure 1:**
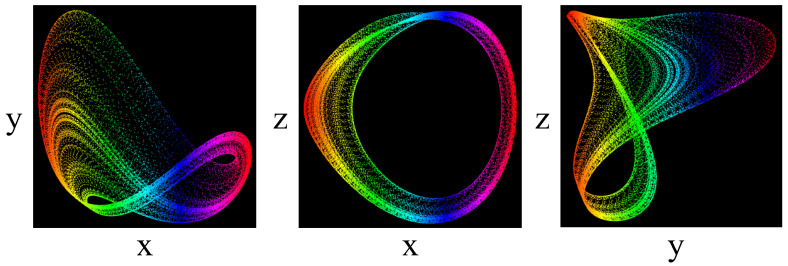
Different projections of the chaotic attractor of system (1) with the initial conditions (−1.8, −1.5, −2.5).

This system is in the chaotic state when a=4.0, b=1.0, c=1.0, d=1.0, e=1.0, f=4.0 and g=1.0. Different projections of the phase portrait for this system are plotted in Figure 1, which shows its strange attractor in 2D state spaces. System (1) is a new offset-boostable one [1,2,3,4] in which the variable z can be boosted with a direct constant in the first dimension.

This system has fixed-points in every $\left(x^{*},y^{*},z^{*}\right)$ which satisfy the following equation,

(2) $$\{\begin{array}{c} \dot{x}=0\\ \dot{y}=0\\ \dot{z}=0 \end{array}\to \{\begin{array}{l} 0=z\\ 0=x^{2}+y^{2}-4\\ 0=-4x-x^{2}+y^{2} \end{array}$$

According to Equation (2), the system (1) has two equilibria in A=(0.7321,3.4641,0) and B=(0.7321,−3.4641,0). The Jacobian matrix of the system (1) is

(3) $$J=\left[\begin{array}{ccc} 0 & 0 & 1\\ 2x & 2y & 0\\ -4-2x & 2y & 0 \end{array}\right]$$

and the corresponding eigenvalues for *A* and *B* are

(4) $$A:\{\begin{array}{l} \lambda_{1}=3.9784\\ \lambda_{2,3}=-0.12798\pm i2.5428 \end{array}\;B:\{\begin{array}{l} \lambda_{1}=-3.9784\\ \lambda_{2,3}=0.12798\pm i2.5428 \end{array}$$

Therefore, both equilibria are saddle-foci. Thus, the attractor is self-excited.

## 3. Bifurcation and Entropy Analysis

### 3.1. Bifurcation Analysis

**Figure 2:**
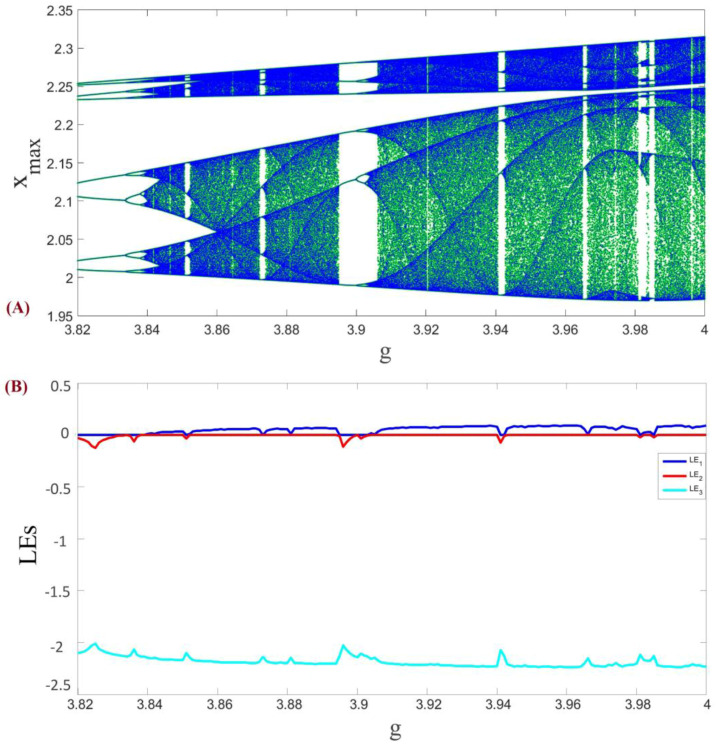
(**A**) Bifurcation diagram of the system (1) with respect to parameter g, and (**B**) Lyapunov exponents of the system (1) with respect to parameter g.

In this part, we investigate the behaviors of the system (1) with respect to changing parameter g. In part (A) of Figure 2 the bifurcation diagram of the system is shown and in part (B) of this figure Lyapunov exponents can be observed. It is important to be careful about numerical calculation of Lyapunov exponents, since improper use of usual methods may cause some issues [14,15,73,74,75,76,77]. We have used the algorithm of [78] for computation of Lyapunov exponents.

As can be seen in Figure 2A, changing parameter g causes a familiar period doubling route to chaos. In addition, positive values of the Lyapunov exponents in Figure 2B show that the underlying system is the chaotic system.

### 3.2. Entropy Analysis

There are many techniques to evaluate the system complexity from data. One of the most famous method which had been used since 1991 is Approximate Entropy (ApEn) [79]. ApEn can be applied to short and noisy data with outliers [80]. Therefore, many systems can be categorized by means of complexity [81]. Consider the *N* data sample u(1), u(2), …, u(N) with the vector sequence $x\left(1\right),\;x\left(2\right),\ldots ,x\left(N-m+1\right)\in R^{m}$ which can be defined as:

(5) x(i)=[u(i), u(i+1), …, u(i+m−1)]

where *m* is an integer and determines the dimension of x(i) as the length of compared run of data. Then, for each i in the 1≤i≤N−m+1, the following equation is defined:

(6) $$c_{i}^{m}\left(r\right)=\frac{J}{N-m+1}$$

(7) d[x(i),x(j)]≤r , 1≤j≤N−m+1

(8) $$d\left[x\left(i\right),x\left(j\right)\right]=\underset{k=1,2,\ldots ,D}{\max}\left(\left|u\left(i+k-1\right)-u\left(j+k-1\right)\right|\right)$$

where *J* is the number of correct vectors in Equation (7), the number of vectors that the distance (infinity norm or maximum norm) between them and x(i) is lower than *r*, and *r* is also a tolerance threshold that is defined by the product of a constant *C* to the standard deviation of data.

(9) r=C×std(u(t)) 0.1≤C≤0.2

Then, the *ApEn* can be written as:

(10) $$\phi^{m}\left(r\right)=\frac{\sum_{i=1}^{N-m+1}logC_{i}^{m}\left(r\right)}{N-m+1}$$

(11) $$ApEn\left(m,r\right)=\underset{N\to \infty }{\lim}\left[\phi^{m}\left(r\right)-\phi^{m+1}\left(r\right)\right]$$

The estimation of Equation (11) for *N* data sample is as follows,

(12) $$ApEn\left(m,r,N\right)=\phi^{m}\left(r\right)-\phi^{m+1}\left(r\right)$$

**Figure 3:**
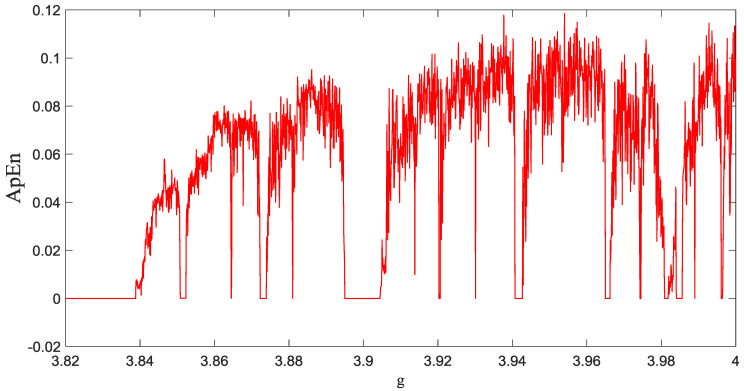
ApEn of the system (1) with respect to parameter g.

It can be derived that the *ApEn* values determine the similarity between chosen window and the sliding window of the data. Therefore, m determines the length of the window to be compared, and *r* is the tolerance threshold for accepting similar pattern between two windows. Figure 3 represents the *ApEn* diagram of the system (1) with respect to parameter g.

## 4. Real Circuit Design of the New Chaotic System as a Mobile RNG and Its Application for Signal Encryption

Random number generator (RNG) algorithms produce a sequence of numbers with properties of randomness and they are a research subject since a few decades. Chaotic systems are commonly used in the random numbers generation algorithms because they are complex and very sensitive. In this section, a mobile RNG design is implemented based on the introduced chaotic system (1) and then signal encryption application is realized with the RNG.

The micro-computer based mobile RNG can be used in many fields especially in encryption studies with low cost and high performance. It is aimed at encryption of multimedia data (audio, image, video, text etc.) with the realized mobile RNG to be flexible and user friendly.

**Figure 4:**
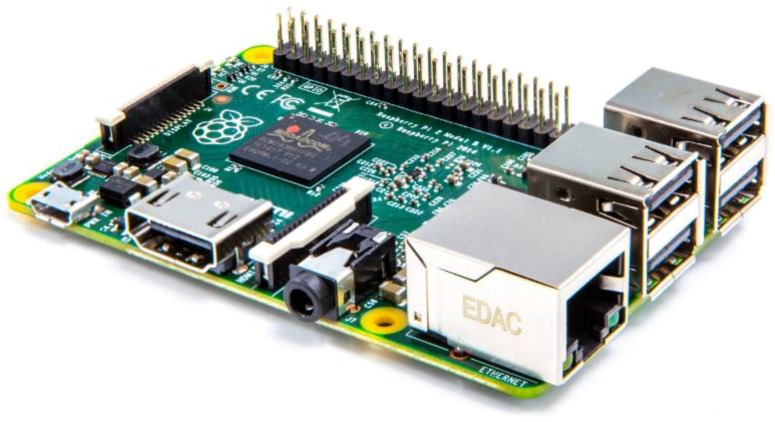
The general outlook of “Raspberry Pi 3”.

As far as we know, random number generators require high cost hardware like computers and FPGA in order to successfully pass the universal tests [82,83,84,85,86]. In this paper, the design of a microcomputer-based mobile RNG and a signal encryption application with the designed RNG is realized without needing hardware such as FPGA, computers, etc. Therefore, “Raspberry Pi 3” is used here as hardware which supports 64-bit processing capability. Since the “Raspberry Pi 3” card has 64-bit processing capability, it can generate very sensitive decimal numbers; thus, randomness of these generated numbers is very high. BCM2837 SoC (system-on-chip) 64-bit ARMv8 quad core Cortex A53 processor running @1.2GHz produced by Broadcom is available on the card. The general view of “Raspberry Pi 3” is as given as in Figure 4.

Our proposed circuit is used as an entropy source for RNG. Then, the NIST-800-22 tests are performed on random numbers to evaluate the performance of the designed RNG. In the next step, a signal encryption application is realized as an example application in “Raspberry Pi 3”. Also, an electronic circuit implementation of the chaotic circuit is done in OrCAD-PSpice and on the oscilloscope.

### 4.1. Micro-Computer-Based Mobile RNG Design

As before mentioned, the “Raspberry Pi 3” board is used as a micro-computer for RNG design and encryption application. The chaotic system of (1) is also utilized in the RNG design. The RNG design steps are given in Algorithm 1 as a pseudo code.

**Algorithm 1.** Mobile RNG design algorithm pseudo code.1:  **Start**2:  Entering parameters and initial condition of the chaotic system3:  Determination of the value of ∆*h*4:  Sampling with determination ∆*h* value5:  **while** (least 1 M. Bit data) do6:   Solving the chaotic system with RK47:   Convert float to binary number (32 bit)8:   Select the bits (LSB-16 bit) from 32 bit binary number9:  **end while**10: The implementation of NIST Tests for 1 M. Bit data11: **if** test results == pass then12:   Successful results (Ready tested 1 M. Bit data)13:   RNG applications (Cryptology, data hiding, watermarking, etc.)14: **else** (test results == false)15: return the previous steps and generate bits again16: **end if**17: **End**

After entering parameters and initial condition of the system (1), the outputs are discretized with the RK4 differential equation solving method. Then, float numbers are obtained and converted into 32 bits binary numbers. Later, the RNG design is executed with obtained binary numbers. The last 16 bits of the outputs (*x*, *y* and *z* variables) are used in the design. The NIST-800-22 statistical tests are also used to prove the success of the RNG design [87]. The NIST-800-22 tests consist of 16 different tests such as monobit, serial and discrete Fourier transform tests. The *p*-values of the test should be greater than 0.001 in order to be counted as successful in NISTS-800-22 tests.

Our experiments show that the random numbers generated from *x*, *y* and *z* outputs successfully passed all the tests with the last 16 bits. The NIST-800-22 tests results are given in Table 1. The ready tested random numbers that pass all of the NIST-800-22 tests can be used in applications that require high security such as cryptology, data hiding, watermarking, etc.

**Figure 5:**
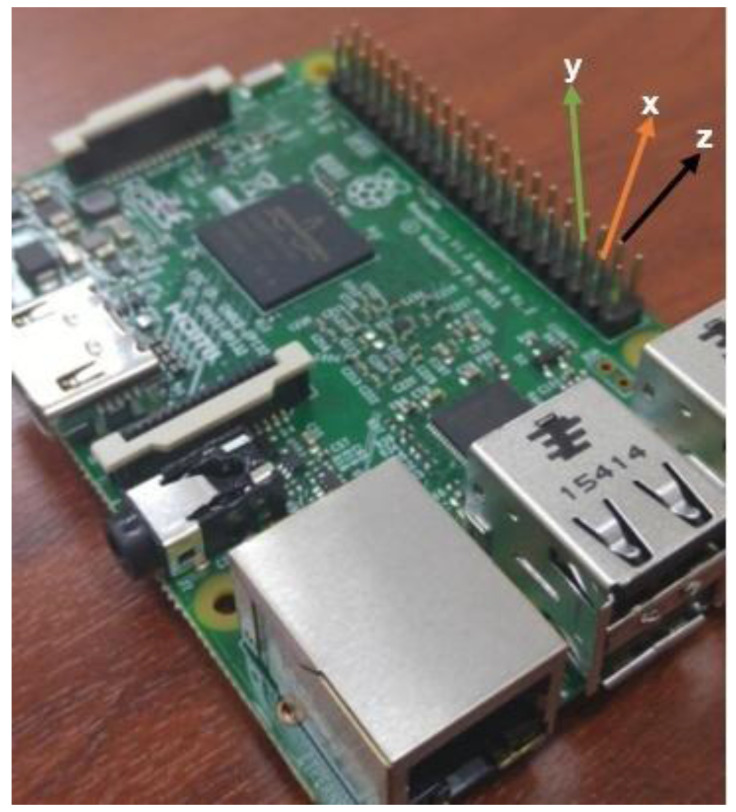
Pins of *x*, *y* and *z* for chaotic system outputs from “Raspberry Pi 3”.

To obtain the random numbers, the pins *x*, *y*, and *z* GPIO (General purpose input/output) are utilized as shown in Figure 5. They are the 37th pin for *x* output, the 35th pin for *y* output, and the 38th pin for *z* output from “Raspberry Pi 3”.

**Figure 6:**
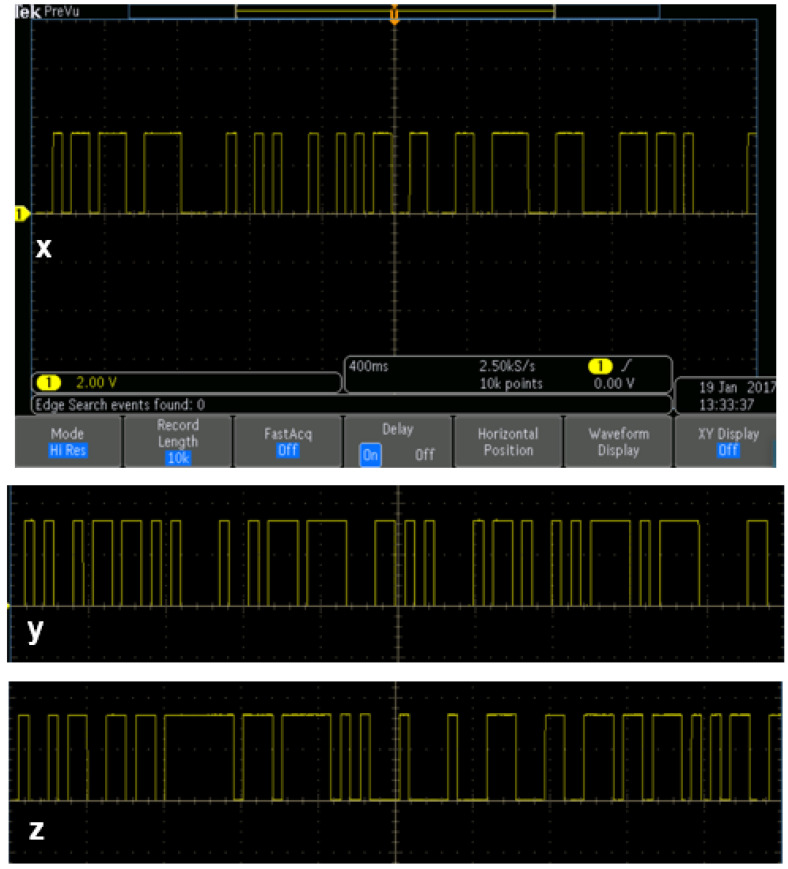
*x*, *y* and *z* outputs on the oscilloscope (first 50 bits).

The generated *x*, *y* and *z* outputs (first 50 bits) are shown in Figure 6 as real-time oscilloscope outputs. The 35th, 37th, and 38th GPIO pins give *x*, *y* and *z* outputs in Figure 5, respectively. They are used for real-time oscilloscope outputs.

### 4.2. Signal Encryption Application Using “Raspberry Pi 3”

**Figure 7:**
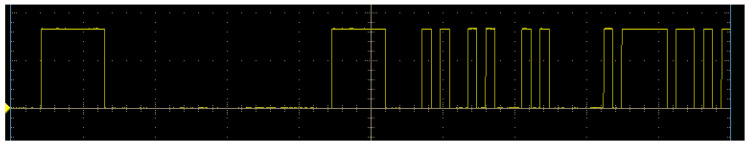
Original signal data (first 50 bits).

In this section, a signal encryption application with RNG that was generated from the proposed chaotic system is realized in “Raspberry Pi 3”. The steps of the encryption and decryption process are given in Algorithm 2. In the encryption application, a signal that consists of 512 bits is used and shown (first 50 bits) in Figure 7 as the real-time oscilloscope outputs.

**Algorithm 2.** Chaos based encryption and decryption algorithm pseudo code.1:  **Start**2:  Getting ready to test random numbers for keys3:  Getting signal data to be encrypted4:  **for**
*i* = 1 for all original data5:   random number bit **xor** original data bit6:  **end**
7:  Encrypted data 8:  **for**
*i* = 1 for all encrypted data9:   random number bit **xor** encrypted data bit10:  **end**11: Decrypted data12: **End**

**Figure 8:**
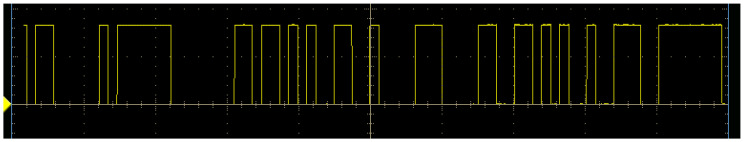
Encrypted signal data (first 50 bits).

For the encryption process, the ‘XOR’ operator is used. Figure 8 shows the first 50 bits of the encrypted signal as real-time oscilloscope outputs. Since the encryption process is performed for each bit, the size of the encrypted data is also 512.

**Figure 9:**
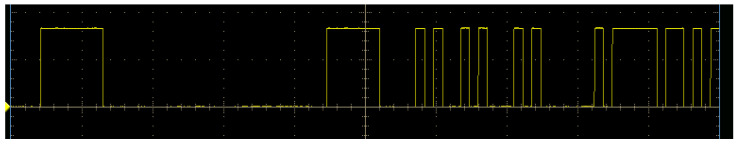
Decrypted Signal Data (first 50 bits).

The same keys generated from the chaotic system are needed for decryption. With these keys, the original data can be obtained, again. The first 50 bits of the decrypted signal are shown in Figure 9 as the real-time oscilloscope outputs. As can be seen, comparing Figure 7 and Figure 9, for the first 50 bits, there is no deformation.

In the implemented method, a cryptoanalyser who wants to crack the encrypted data must know exactly all of the parameters and initial values of the chaotic system used in the encryption. Also, encrypted data will be not decrypted without “Raspberry Pi 3”.

### 4.3. Electronic Circuit Implementation of the Chaotic System in OrCAD-PSpice and on the Oscilloscope

**Figure 10:**
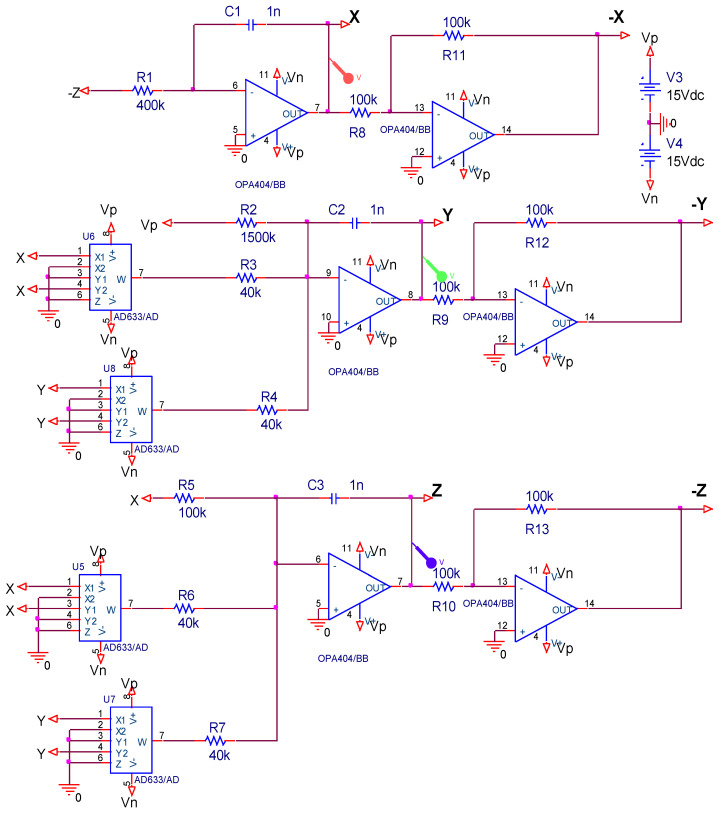
The electronic circuit schematic of system (1).

**Figure 11:**
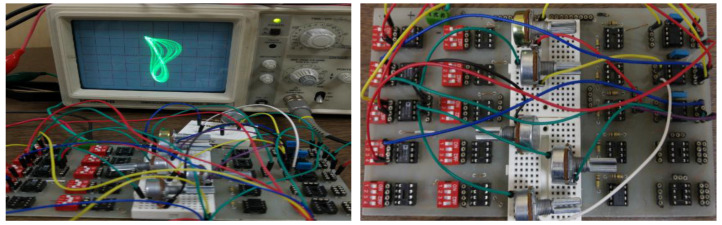
The experimental circuit of the chaotic circuit and the phase portraits of system (1) on the oscilloscope.

In this part, we design an electronic circuit based on system (1) in OrCAD-PSpice (Figure 10) and on the board (Figure 11). The circuit includes simple electronic elements such as resistors, multipliers, capacitor, and opamps. Note that PSPICE simulation of chaotic circuits is quite trivial. In the literature, such systems are implemented with integrated circuit technology [88].

**Figure 12:**
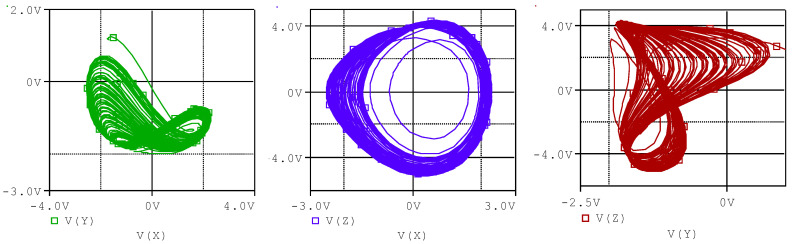
The phase portraits of the system (1) in ORCAD-Pspice.

**Figure 13:**
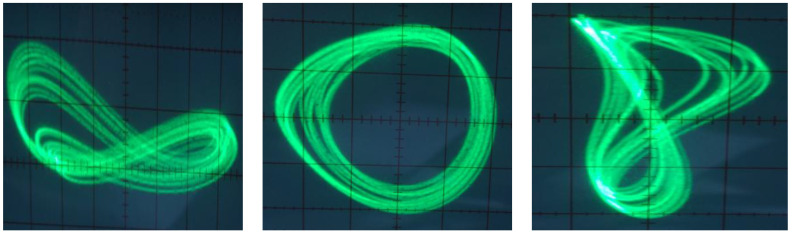
The phase portraits of system (1) on the oscilloscope.

The OrCAD-PSpice simulation outputs, which are two-dimensional phase portraits of the chaotic system, are seen in Figure 12 and Figure 13, respectively. As can be seen from the ORCAD-PSpice outputs in Figure 12 and oscilloscope outputs in Figure 13, the results are similar.

## 5. Parameter Estimation of the Chaotic System

In this section, we introduce the parameter estimation method used for the chaotic circuit. This method utilizes a cost function which was adopted for the chaotic systems. The cost function of the parameter estimation method is based on a similarity metric using a parametric model of strange attractors in the state space. It was shown that this cost function could yield better results than the conventional error-based cost function over the time-domain [71]. The time-independent property of the state space is a sufficient reason to use this cost function because the state space can show complex behaviors of the strange attractor of chaotic systems [89].

As before mentioned, the utilized cost function is based on the attractor modeling; therefore, we need a model to represent the distribution of the attractor points in the state space. As a smooth parametric model, a Gaussian mixture model (GMM) can model the chaotic attractor geometry in the state space [67]. The GMM is a parametric probability density function represented by a weighted sum of Gaussian component densities [90]. It can model the distribution of the attractor points in the state space based on its powerful characteristics [91]. So far, metrics such as Kullback–Leibler divergence (also called relative entropy) were defined to measure distance between GMMs [90]. In addition, similarity-based metrics such as likelihood functions have been used to measure distance between a time series and a GMM. This idea was recently used as phone classification methods by parametric models of the distribution points of the speech signal in a high-dimension domain named RPS [67,68,69].

The GMMs have also been used for parameter estimation of some chaotic systems [70,71]. They were utilized similar to the task of the phone classification method. Suppose we have a chaotic system with a known model and its trajectory was recorded. We can then generate a GMM for the strange attractor of the chaotic system in the state space. Utilizing a distance-like metric over a likelihood function, we can compute dissimilarity between the learned GMM model of the real system attractor (with unknown parameters) and a distribution of a new attractor obtained by a system’s model (with known parameters) in the state space to complete the parameter estimation method. Therefore, the score of the distance-based metric will be equal to the cost function of the parameter estimation method.

### 5.1. The GMM Computation as a Cost Function

A GMM with M mixtures is a weighted sum of M individual Gaussian densities. Each Gaussian density as a component of the GMM is represented by three main factors, mixture weight, mean vector, and covariance matrix. Therefore, they can be shown by a set of parameters, *λ*, as follows,

(13) $$\lambda =\left\{w_{m},{µ}_{m},\Sigma_{m}\right\},\;m=1,\ldots ,M\;p\left(v|\lambda \right)=\overset{M}{\underset{m=1}{\sum }}w_{m}\frac{1}{{\left(2\pi \right)}^{D/2}}\frac{1}{{\left|\Sigma_{m}\right|}^{1/2}}\exp\left\{\frac{-1}{2}{\left(v-{µ}_{m}\right)}^{T}\Sigma_{m}{}^{-1}\left(v-{µ}_{m}\right)\right\}$$

and p(v|λ) is the conditional probability of a *D*-dimensional single observation vector ***v*** given the GMM of λ. The p(v|λ) can show a likelihood score. It expresses how probable the observed vector *v* is for the GMM of λ. In Equation (13) |.| is the determinant operator, exp(.) denotes the exponential function, and *M* is the number of mixtures (Gaussian components). In addition, for *m*-th mixture, $w_{m}\in \left[0,1\right]$ is an scalar and named *m*-th mixing coefficient or mixture weight, ${µ}_{m}$ is the *m*-th *D*-dimensional mean vector, and $\Sigma_{m}$ is the *m*-th *D* × *D* covariance matrix. The mean vector and covariance matrix of a Gaussian component can show the center and the shape of points distribution around those of the component. It should be noted that the mixing coefficients $w_{m}$ are constrained to sum to 1, i.e., $\sum_{m=1}^{M}w_{m}=1$.

As a problem which depends on the complexity of the data distribution, there is no analytical solution to determine the optimum number of GMM mixtures, *M*, needed for modeling of the attractors. Therefore, it is common to use a trial-and-error method to choose an adequate value of *M*. In our attractor modeling problem, to obtain a proper GMM model of the attractor in the state space, we evaluate some values of *M*. Generally, we need a higher value of *M* for attractor modeling if it has a very complex dynamic in the state space. One should note that while a higher number of mixtures can increase the performance of the cost function, it also increases the computational cost.

To find the similarity score between the attractor of a real system and the state space points of a specific model obtained from a chaotic system with known parameters (for example chaotic system (1)), the likelihood score can be calculated. Therefore, the parameter estimation of a known chaotic system with unknown parameters can be performed using the following two phases; a learning phase, here named “phase A”, which includes fitting the GMM to the attractor of the real system, and an evaluation phase, named “phase B”, to select the best values of parameters for the known chaotic model which causes the maximum similarity score or equally minimum distance score (cost function) over the learned GMM. Following are those phases in details:

### 5.2. Phase A

The first phase of the parameter estimation approach is the learning phase to find the GMM parameters, λ in Equation (13). The GMM learns the attractor’s distribution of a real system, e.g., a chaotic circuit. Suppose $S=\left\{s_{1},s_{2},\ldots ,s_{N}\right\}$ is an N×D matrix consisting of N-samples of the time series of the real data in the D-dimensional state space. Therefore, each sample is a *D*-dimensional observation vector. To find the GMM parameters, an iterative expectation-maximization (EM) algorithm is utilized as follows:

#### 5.2.1. Initialization Step

Initialize the mean vector ${µ}_{m}$, covariance matrix $\Sigma_{m}$ and mixing coefficients $w_{m}$ in Equation (13) and evaluate the initial value of the logarithm of the likelihood score obtained from the input time series as follows,

(14) $$logp\left(S|\lambda \right)=\overset{N}{\underset{n=1}{\sum }}log\left(p(s_{n}|\lambda )\right)$$

#### 5.2.2. Expectation Step

Evaluate values of $r\left(s_{i},m\right)$, named responsibility of *i*-th sample of ***S*** given the *m*-th Gaussian component, using the current values of the GMM parameters:

(15) $$r\left(s_{i},m\right)=\frac{w_{m}\frac{1}{{\left(2\pi \right)}^{D/2}}\frac{1}{{\left|\Sigma_{m}\right|}^{1/2}}exp\left\{\frac{-1}{2}{\left(s_{i}-{µ}_{m}\right)}^{T}\Sigma_{m}{}^{-1}\left(s_{i}-{µ}_{m}\right)\right\}}{\sum_{j=1}^{M}w_{j}\frac{1}{{\left(2\pi \right)}^{D/2}}\frac{1}{{\left|\Sigma_{j}\right|}^{1/2}}exp\left\{\frac{-1}{2}{\left(s_{i}-{µ}_{j}\right)}^{T}\Sigma_{j}{}^{-1}\left(s_{i}-{µ}_{j}\right)\right\}}$$

#### 5.2.3. Maximization Step

Re-estimate the parameters of the GMM utilizing the estimated values of the responsibilities as follows:

(16) $$N_{m}=\sum_{i=1}^{N}r\left(s_{i},m\right)$$

(17) $${µ}_{m}=\frac{1}{N_{m}}\sum_{i=1}^{N}r\left(s_{i},m\right)s_{i}$$

(18) $$\Sigma_{m}=\frac{1}{N_{m}}\sum_{i=1}^{N}r\left(s_{i},m\right)\left(s_{i}-{µ}_{m}\right){\left(s_{i}-{µ}_{m}\right)}^{T}$$

(19) $$w_{m}=\frac{N_{m}}{N}$$

#### 5.2.4. Likelihood Computation Step

Evaluate the logarithm of the likelihood score in Equation (14) and check for convergence criterion. If the convergence criterion is not satisfied, return to Section 5.2.2.

### 5.3. Phase B

The second phase is finding the best parameters of the known model of the chaotic system (with unknown parameters) using the learned GMM in the phase A. Here, the search space will be formed from a set of acceptable values of the model parameters. Now we suppose that the values of the parameters *a*&*b* of the system (1) are unknown. Then, for each pair of parameters (a,b), the chaotic system of (1) will be simulated, and a trajectory $T\left(a,b\right)=\left(t_{1},t_{2},t_{3},\ldots ,t_{K}|a,b\right)$ with K samples will be obtained where each $t_{K}$ is *D*-dimensional measured data in the state space. Finally, using an average point-by-point log-likelihood score obtained from the learned GMM, λ, a similarity-based score is computed as follows,

(20) $$log\left(p(T^{\left(a,b\right)}|\lambda )\right)=\frac{1}{K}\overset{K}{\underset{K=1}{\sum }}log\left(p(t_{k}|\lambda )\right)$$

where $T^{\left(a,b\right)}$ is a matrix whose rows are composed from the state space vectors of the system trajectory with the model’s parameters (a,b), and *K* is the number of the state space point. The parameter estimation method of the model is accomplished by computing Equation (20) and selecting the parameters of the model that can obtain the best similarity-based score, which here means the maximum score. If we use the negative of the similarity-based score, then the parameter estimation becomes a cost function minimization. Therefore, the best parameter selection, ${\left(a,b\right)}^{*}$, would be conducted by the following criteria, J(.), based on the negative of mean log-likelihood score,

(21) $${\left(a,b\right)}^{*}=argmin\left\{J\left(\left(a,b\right)\right)\right\}\&\;J\left(\left(a,b\right)\right)=-p\left(T^{\left(a,b\right)}|\lambda \right)$$

Equation (21) shows the utilized cost function and (a,b) is the set of the system parameters (1). Here, λ is the learned GMM of the real system attractor obtained from the phase A. The objective of the parameter estimation method is to determine the parameters of the system, (a,b) when the cost function is minimized to result in the minimum value of J((a,b)). The minimum value of the cost function guarantees the best solution with the proper parameters.

### 5.4. The GMM of Chaotic Circuit

**Figure 14:**
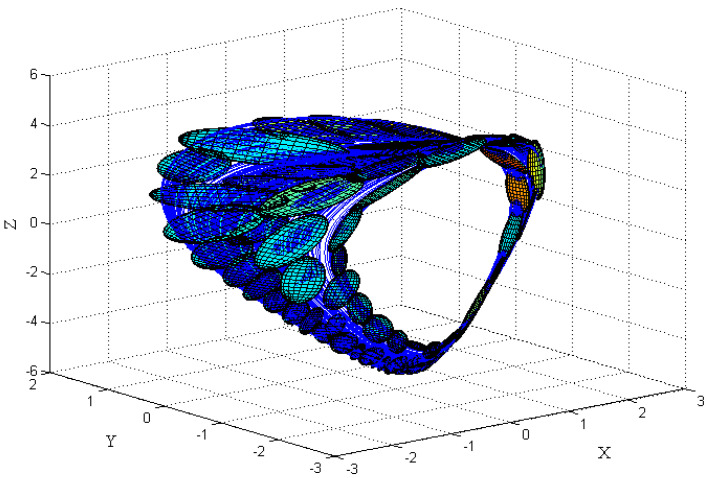
Plot of the attractor and its GMM modeling with M=256 components for the chaotic system (1) with  a=4 & b=1, in the 3-D state space.

Based on the observation vector *v* of the chaotic circuit, in this work, *D* = 3 is selected for the dimension of the state space according to system (1). Using the prepared real training data for the attractor by the chaotic circuit as a real system, the GMM will be specialized in order to model the geometry of that attractor. Figure 14 shows the attractor of the chaotic system in a three-dimensional state space with its GMM modeling using 256 Gaussian components, M=256, where every three-dimensional ellipsoid corresponds to one of the Gaussian components. 

## 6. Optimization Algorithm

There are four major categories to which different kinds of optimization methods belong: Enumerative methods, Calculus-Based methods, Heuristic methods, and Meta-heuristic methods [62]. Meta-heuristic optimization algorithms, which cover a wide range of problems, are becoming more and more popular in engineering applications [92,93]. Nature can be named as one of the most important sources of inspiration for new meta-heuristic algorithms. On this subject, black and white holes in cosmology and Humpback whales in the sea aid in constructing the MVO (Multi-Verse Optimizer) and WOA (Whale Optimization Algorithm) meta-heuristic algorithm [94,95]. We now introduce these methods:

### 6.1. The Whale Optimization Algorithm

The WOA algorithm is based on the hunting behavior of Humpback whales which can encircle the recognized location of prey. The WOA algorithm assumes that the current best candidate solution is the target prey or is close to the optimum. The next step is about the attacking strategy which is the bubble-net strategy. Putting it all together, the proposed WOA method includes three major steps in the simulation: the search for prey, encircling prey, and the bubble-net foraging behavior of humpback whales. For complete details see [94].

### 6.2. Multi-Verse Optimizer: A Nature-Inspired Algorithm for GlobalOptimization

Another novel nature-inspired algorithm is Multi-Verse Optimizer (MVO). Cosmology (white hole, black hole, and wormhole) is the main inspiration of this algorithm. As mentioned before, every search process in the optimization algorithm consists of two phase: exploration and exploitation. The MVO supports this by white and black holes in order to respond to the exploration phase and wormholes for the exploitation phase. Further details are described in [95].

### 6.3. Experimental Results

In this section, some simulations are done to investigate the acceptability of the parameter estimation method of the chaotic circuit. We have used a fourth-order Runge-Kutta method with a step size of 10 ms and a total of 30,000 samples corresponding to a time of 300 s. Here, we assume that the original chaotic system of (1) should be estimated by minimization of the GMM-based cost function.

**Figure 15:**
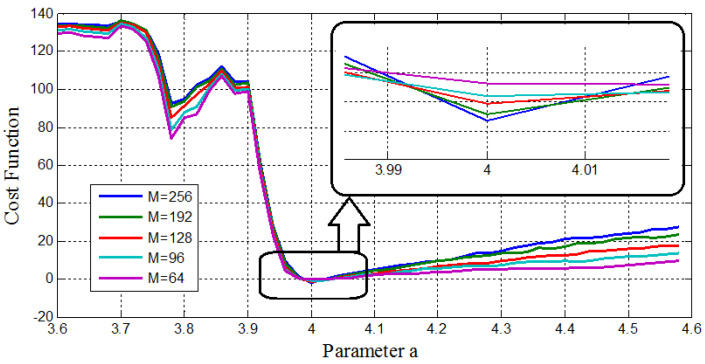
Cost function versus parameter *a*, with different number GMM components (M) for the 1D parameter estimation method.

**Figure 16:**
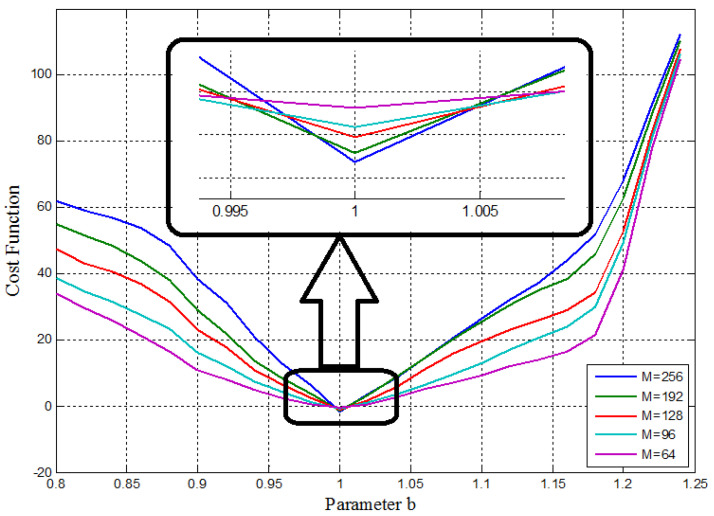
Cost function versus parameter *b*, with different number of GMM components (M) for the 1D parameter estimation method.

First, using some 1D parameter estimation methods, different number of the GMM’s components, M=(64,96,128,192,256), are used to show the sufficiency of the cost function. The experimental results of the cost function versus the values of the parameters *a*&*b* are depicted in Figure 15 and Figure 16, respectively. 

As can be seen, all of the cost functions show convex functions around the desired point. Therefore, they are acceptable for the parameter estimation methods. Specifically, they show the effect of changing the parameter of the model as a monotonically trend along with a global minimum at the exact expected value of the desired parameters (a=4.00, b=1.00). Therefore, the GMM—based cost function has the desired ideal properties for the parameter estimation problem. Moreover, Figure 15 and Figure 16 show the effect of increasing the number of GMM components, *M*, used in the GMM modeling. In this case, M=256 represents better performance to identify the parameters *a*&*b*.

**Figure 17:**
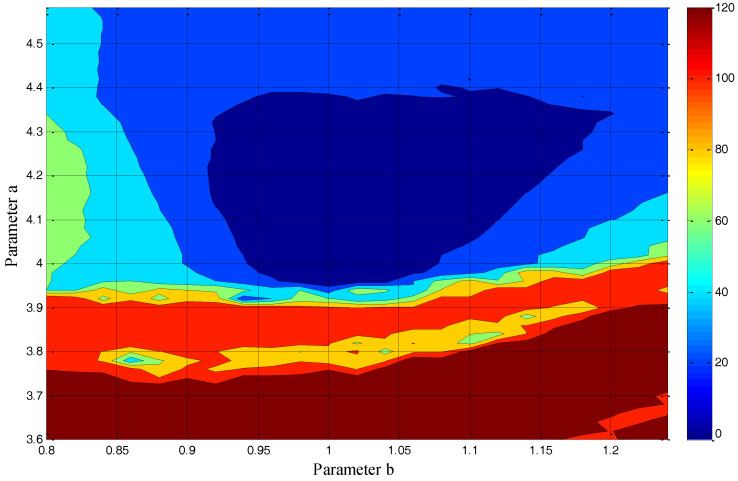
The contour plot of the GMM-based cost function for the introduced chaotic system (M=256) along with variations in the parameters, *a*&*b*.

**Figure 18:**
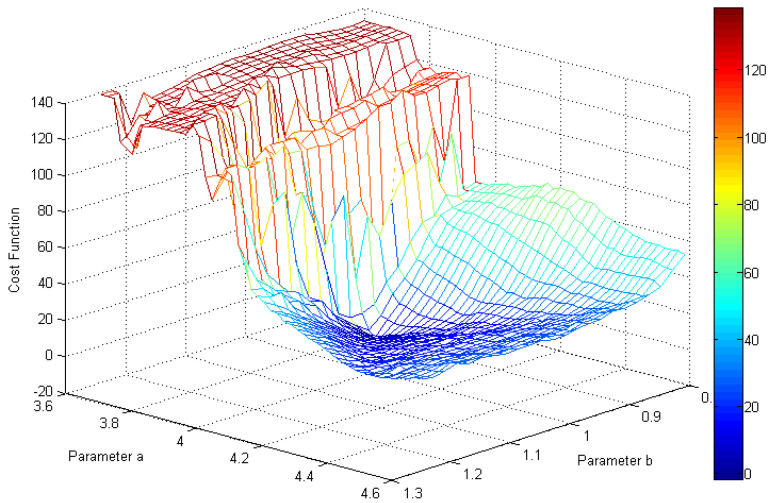
The “cost surface” of the GMM-based cost function for the introduced chaotic system (M=256) along with variations in the parameters, *a*&*b*.

In Figure 17 and Figure 18, a contour plot of the cost function and its “cost surface” are respectively shown for the chaotic system (1) with M=256 along with variation in the parameters, *a*&*b*. They show dissimilarity between the real system attractor and each model attractor for a 2D parameter estimation problem. The minimum value of the point on those plots gives the parameters for the best model.

As can be seen in Figure 17 and Figure 18, the global minimum of the cost function is in the right place (a=4.00 and b=1.00). Furthermore, the surface of the cost function is almost convex near the best parameters, which makes it an easy case for any optimization approach that moves downhill.

**Figure 19:**
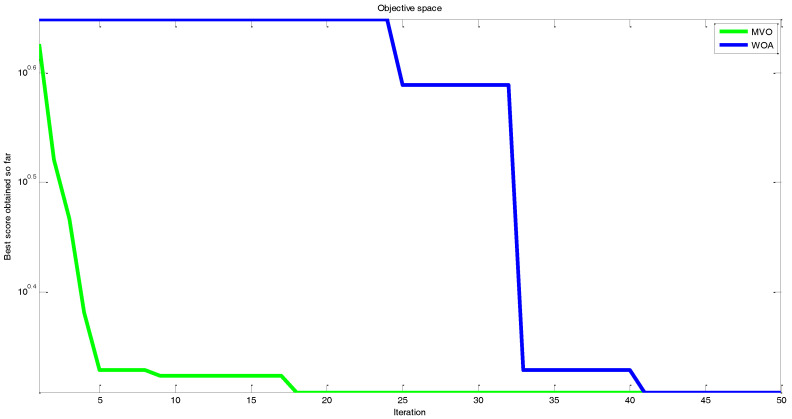
Comparison between the performances of the MVO and WOA optimization algorithm.

In order to examine the efficiency of the cost function in the parameter estimation, two mentioned meta-heuristic optimization methods are applied. All the basic parameters, such as maximum number of iterations (50) and number of search agent (25), are the same in both algorithms. For further details about the algorithms and their particular parameters, see [96]. Comparison between the performances of MVO and WOA optimization algorithm is shown in Figure 19.

**Figure 20:**
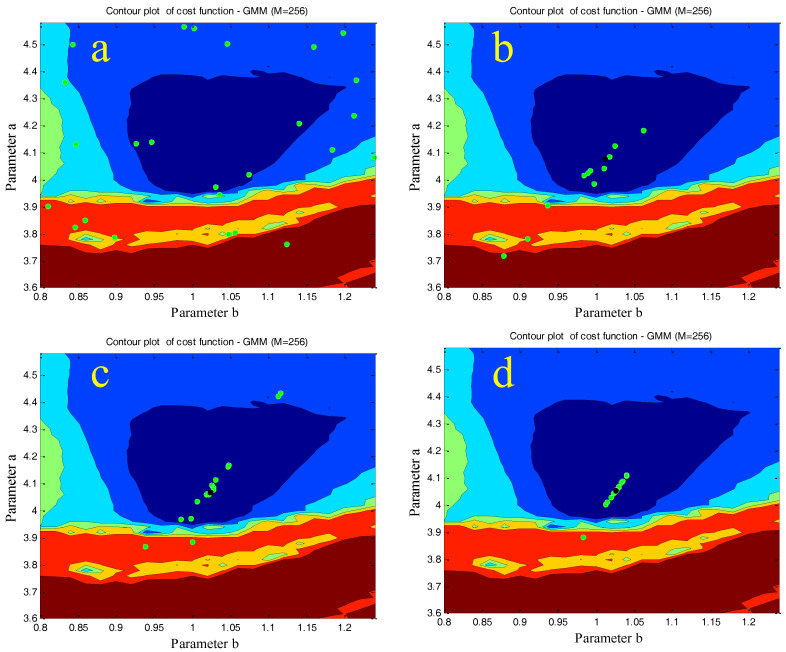
Process of finding the best parameters using the WOA algorithm. (**a**–**d**) represent the first, 10th, 20th, and 30th iteration, respectively.

Based on the results of Figure 19, the MVO optimization method showed a superior performance in comparison with the WOA algorithms. In addition, Figure 20 represents the process of finding the best parameters using the WOA algorithm performed once for every 10 iterations. As can be seen, the individuals converge to the optimum area (a=4.00 and b=1.00).

## 7. Conclusions

In this paper, a new chaotic system has been investigated carefully through bifurcation, the largest Lyapunov exponent, ApEn, and stability analysis. Then, an engineering application of that system was proposed using a random number generator and its signal encryption application. After that, a GMM-based cost function was utilized in the parameter estimation of the chaotic circuit designed from the chaotic system. The cost function was based on the minimization of dissimilarity between the phase portrait obtained from the real system and that obtained from the model of the chaotic system. In order to minimize the cost function and to obtain the correct parameters, we used two new efficient optimization methods, the Whale Optimization Algorithm (WOA), and Multi-Verse Optimizer (MVO) algorithm. The MVO optimization method showed superior performance in comparison with the WOA algorithm.

## Figures and Tables

**Table 1:** RNG NIST-800-22 tests for *x*, *y* and *z* outputs.

Statistical Tests	*p*-Value-*x* (X_16bit)	*p*-Value-*y* (Y_16bit)	*p*-Value-*z* (Z_16bit)	Result
Frequency (Monobit) Test	0.5741	0.2209	0.9904	Successful
Block-Frequency Test	0.5692	0.2711	0.4011	Successful
Cumulative-Sums Test	0.6255	0.1218	0.4619	Successful
Runs Test	0.7012	0.1846	0.5313	Successful
Longest-Run Test	0.6207	0.1881	0.6901	Successful
Binary Matrix Rank Test	0.4378	0.9036	0.9755	Successful
Discrete Fourier Transform Test	0.0796	0.5819	0.6931	Successful
Non-Overlapping Templates Test	0.1685	0.0011	0.0803	Successful
Overlapping Templates Test	0.8824	0.1699	0.5441	Successful
Maurer’s Universal Statistical Test	0.5665	0.3602	0.8932	Successful
Approximate Entropy Test	0.1364	0.7072	0.6264	Successful
Random-Excursions Test (*x* = −4)	0.9005	0.3467	0.6683	Successful
Random-Excursions Variant Test (*x* = 9)	0.5249	0.9845	0.5880	Successful
Serial Test-1	0.1784	0.6299	0.5716	Successful
Serial Test-2	0.5467	0.4709	0.7633	Successful
Linear-Complexity Test	0.7039	0.3601	0.2000	Successful

## References

[1] Li C., Sprott J.C. (2016). Variable-boostable chaotic flows. Opt.-Int. J. Light Electron Opt..

[2] Li C., Sprott J.C., Akgul A., Iu H.H., Zhao Y. (2017). A new chaotic oscillator with free control. Chaos Interdiscip. J. Nonlinear Sci..

[3] Jafari M.A., Mliki E., Akgul A., Pham V.-T., Kingni S.T., Wang X., Jafari S. (2017). Chameleon: The most hidden chaotic flow. Nonlinear Dyn..

[4] Li C., Sprott J.C., Xing H. (2016). Hypogenetic chaotic jerk flows. Phys. Lett. A.

[5] Tlelo-Cuautle E., Carbajal-Gomez V., Obeso-Rodelo P., Rangel-Magdaleno J., Nuñez-Perez J.C. (2015). FPGA realization of a chaotic communication system applied to image processing. Nonlinear Dyn..

[6] De la Fraga L.G., Torres-Pérez E., Tlelo-Cuautle E., Mancillas-López C. (2017). Hardware implementation of pseudo-random number generators based on chaotic maps. Nonlinear Dyn..

[7] Pano-Azucena A.D., de Jesus Rangel-Magdaleno J., Tlelo-Cuautle E., de Jesus Quintas-Valles A. (2017). Arduino-based chaotic secure communication system using multi-directional multi-scroll chaotic oscillators. Nonlinear Dyn..

[8] Valtierra J.L., Tlelo-Cuautle E., Rodríguez-Vázquez Á. (2017). A switched-capacitor skew-tent map implementation for random number generation. Int. J. Circuit Theory Appl..

[9] García-Martínez M., Ontañón-García L., Campos-Cantón E., Čelikovský S. (2015). Hyperchaotic encryption based on multi-scroll piecewise linear systems. Appl. Math. Comput..

[10] Tlelo-Cuautle E., Rangel-Magdaleno J., Pano-Azucena A., Obeso-Rodelo P., Nuñez-Perez J.C. (2015). FPGA realization of multi-scroll chaotic oscillators. Commun. Nonlinear Sci. Numer. Simul..

[11] Danca M.-F., Kuznetsov N. (2017). Hidden chaotic sets in a Hopfield neural system. Chaos Solitons Fractals.

[12] Danca M.-F., Kuznetsov N., Chen G. (2017). Unusual dynamics and hidden attractors of the Rabinovich–Fabrikant system. Nonlinear Dyn..

[13] Kuznetsov N., Leonov G., Yuldashev M., Yuldashev R. (2017). Hidden attractors in dynamical models of phase-locked loop circuits: Limitations of simulation in MATLAB and SPICE. Commun. Nonlinear Sci. Numer. Simul..

[14] Leonov G., Kuznetsov N., Mokaev T. (2015). Hidden attractor and homoclinic orbit in Lorenz-like system describing convective fluid motion in rotating cavity. Commun. Nonlinear Sci. Numer. Simul..

[15] Leonov G.A., Kuznetsov N.V., Mokaev T.N. (2015). Homoclinic orbits, and self-excited and hidden attractors in a Lorenz-like system describing convective fluid motion. Eur. Phys. J. Spec. Top..

[16] Sharma P., Shrimali M., Prasad A., Kuznetsov N., Leonov G. (2015). Control of multistability in hidden attractors. Eur. Phys. J. Spec. Top..

[17] Sharma P.R., Shrimali M.D., Prasad A., Kuznetsov N., Leonov G. (2015). Controlling Dynamics of Hidden Attractors. Int. J. Bifurc. Chaos.

[18] Leonov G., Kuznetsov N., Vagaitsev V. (2011). Localization of hidden Chua’s attractors. Phys. Lett. A.

[19] Leonov G., Kuznetsov N., Vagaitsev V. (2012). Hidden attractor in smooth Chua systems. Phys. D Nonlinear Phenom..

[20] Leonov G.A., Kuznetsov N.V. (2013). Hidden attractors in dynamical systems. From hidden oscillations in Hilbert–Kolmogorov, Aizerman, and Kalman problems to hidden chaotic attractor in Chua circuits. Int. J. Bifurc. Chaos.

[21] Leonov G., Kuznetsov N., Kiseleva M., Solovyeva E., Zaretskiy A. (2014). Hidden oscillations in mathematical model of drilling system actuated by induction motor with a wound rotor. Nonlinear Dyn..

[22] Dudkowski D., Jafari S., Kapitaniak T., Kuznetsov N.V., Leonov G.A., Prasad A. (2016). Hidden attractors in dynamical systems. Phys. Rep..

[23] Tlelo-Cuautle E., de la Fraga L.G., Pham V.-T., Volos C., Jafari S., de Jesus Quintas-Valles A. (2017). Dynamics, FPGA realization and application of a chaotic system with an infinite number of equilibrium points. Nonlinear Dyn..

[24] Pham V.-T., Volos C., Jafari S., Kapitaniak T. (2017). Coexistence of hidden chaotic attractors in a novel no-equilibrium system. Nonlinear Dyn..

[25] Pham V.-T., Kingni S.T., Volos C., Jafari S., Kapitaniak T. (2017). A simple three-dimensional fractional-order chaotic system without equilibrium: Dynamics, circuitry implementation, chaos control and synchronization. AEU Int. J. Electron. Commun..

[26] Pham V.-T., Jafari S., Volos C., Gotthans T., Wang X., Hoang D.V. (2017). A chaotic system with rounded square equilibrium and with no-equilibrium. Opt.-Int. J. Light Electron Opt..

[27] Pham V.-T., Volos C., Gambuzza L.V. (2014). A memristive hyperchaotic system without equilibrium. Sci. World J..

[28] Jafari S., Sprott J.C., Hashemi Golpayegani S.M.R. (2013). Elementary quadratic chaotic flows with no equilibria. Phys. Lett. A.

[29] Pham V.-T., Jafari S., Volos C., Wang X., Hashemi Golpayegani S.M.R. (2014). Is that really hidden? The presence of complex fixed-points in chaotic flows with no equilibria. Int. J. Bifurc. Chaos.

[30] Pham V.-T., Volos C., Jafari S., Wang X. (2014). Generating a novel hyperchaotic system out of equilibrium. Optoelectron. Adv. Mater.-Rapid Commun..

[31] Pham V.-T., Volos C., Jafari S., Wei Z., Wang X. (2014). Constructing a novel no-equilibrium chaotic system. Int. J. Bifurc. Chaos.

[32] Tahir F.R., Jafari S., Pham V.-T., Volos C., Wang X. (2015). A Novel No-Equilibrium Chaotic System with Multiwing Butterfly Attractors. Int. J. Bifurc. Chaos.

[33] Pham V.-T., Jafari S., Kapitaniak T., Volos C., Kingni S.T. (2017). Generating a Chaotic System with One Stable Equilibrium. Int. J. Bifurc. Chaos.

[34] Wang X., Pham V.-T., Jafari S., Volos C., Munoz-Pacheco J.M., Tlelo-Cuautle E. (2017). A new chaotic system with stable equilibrium: From theoretical model to circuit implementation. IEEE Access.

[35] Kingni S.T., Pham V.-T., Jafari S., Woafo P. (2017). A chaotic system with an infinite number of equilibrium points located on a line and on a hyperbola and its fractional-order form. Chaos Solitons Fractals.

[36] Jafari S., Sprott J.C. (2013). Simple chaotic flows with a line equilibrium. Chaos Solitons Fractals.

[37] Pham V.-T., Jafari S., Volos C. (2017). A novel chaotic system with heart-shaped equilibrium and its circuital implementation. Opt.-Int. J. Light Electron Opt..

[38] Pham V.T., Volos C., Kapitaniak T., Jafari S., Wang X. (2017). Dynamics and circuit of a chaotic system with a curve of equilibrium points. Int. J. Electron..

[39] Pham V.-T., Jafari S., Wang X., Ma J. (2016). A Chaotic System with Different Shapes of Equilibria. Int. J. Bifurc. Chaos.

[40] Pham V.-T., Jafari S., Volos C., Giakoumis A., Vaidyanathan S., Kapitaniak T. (2016). A chaotic system with equilibria located on the rounded square loop and its circuit implementation. IEEE Trans. Circuits Syst. II Express Br..

[41] Pham V.-T., Jafari S., Volos C., Vaidyanathan S., Kapitaniak T. (2016). A chaotic system with infinite equilibria located on a piecewise linear curve. Opt.-Int. J. Light Electron Opt..

[42] Jafari S., Sprott J.C., Molaie M. (2016). A simple chaotic flow with a plane of equilibria. Int. J. Bifurc. Chaos.

[43] Jafari S., Sprott J.C., Pham V.-T., Volos C., Li C. (2016). Simple chaotic 3D flows with surfaces of equilibria. Nonlinear Dyn..

[44] Rajagopal K., Akgul A., Jafari S., Karthikeyan A., Koyuncu I. (2017). Chaotic chameleon: Dynamic analyses, circuit implementation, FPGA design and fractional-order form with basic analyses. Chaos Solitons Fractals.

[45] Rajagopal K., Jafari S., Laarem G. (2017). Time-delayed chameleon: Analysis, synchronization and FPGA implementation. Pramana.

[46] Pham V.-T., Wang X., Jafari S., Volos C., Kapitaniak T. (2017). From Wang–Chen System with Only One Stable Equilibrium to a New Chaotic System without Equilibrium. Int. J. Bifurc. Chaos.

[47] Pham V.-T., Volos C., Jafari S., Vaidyanathan S., Kapitaniak T., Wang X. (2016). A chaotic system with different families of hidden attractors. Int. J. Bifurc. Chaos.

[48] Nazarimehr F., Saedi B., Jafari S., Sprott J.C. (2017). Are perpetual points sufficient for locating hidden attractors?. Int. J. Bifurc. Chaos.

[49] Dudkowski D., Prasad A., Kapitaniak T. (2017). Perpetual Points: New Tool for Localization of Coexisting Attractors in Dynamical Systems. Int. J. Bifurc. Chaos.

[50] Faure P., Korn H. (2001). Is there chaos in the brain? I. Concepts of nonlinear dynamics and methods of investigation. C. R. l’Acad. Sci.-Seri. III-Sci..

[51] Korn H., Faure P. (2003). Is there chaos in the brain? II. Experimental evidence and related models. C. R. Biol..

[52] Molaie M., Falahian R., Gharibzadeh S., Jafari S., Sprott J.C. (2014). Artificial neural networks: Powerful tools for modeling chaotic behavior in the nervous system. Front. Comput. Neurosci..

[53] Falahian R., Mehdizadeh Dastjerdi M., Molaie M., Jafari S., Gharibzadeh S. (2015). Artificial neural network-based modeling of brain response to flicker light. Nonlinear Dyn..

[54] Jafari S., Sprott J.C., Hashemi Golpayegani S.M.R. (2016). Layla and Majnun: A complex love story. Nonlinear Dyn..

[55] Aram Z., Jafari S., Ma J., Sprott J.C., Zendehrouh S., Pham V.-T. (2017). Using chaotic artificial neural networks to model memory in the brain. Commun. Nonlinear Sci. Numer. Simul..

[56] Hilborn R.C. (2000). Chaos and Nonlinear Dynamics: An Introduction for Scientists and Engineers.

[57] Jafari S., Hashemi Golpayegani S.M.R., Daliri A. (2013). Comment on ‘Parameters identification of chaotic systems by quantum-behaved particle swarm optimization’ [Int. J. Comput. Math. 86(12) (2009), pp. 2225–2235]. Int. J. Comput. Math..

[58] Jafari S., Hashemi Golpayegani S.M.R., Rasoulzadeh Darabad M. (2013). Comment on “Parameter identification and synchronization of fractional-order chaotic systems” [Commun Nonlinear Sci Numer Simulat 2012; 17: 305–16]. Commun. Nonlinear Sci. Numer. Simul..

[59] Jafari S., Hashemi Golpayegani S.M.R., Jafari A.H., Gharibzadeh S. (2012). Some remarks on chaotic systems. Int. J. Gen. Syst..

[60] He Q., Wang L., Liu B. (2007). Parameter estimation for chaotic systems by particle swarm optimization. Chaos Solitons Fractals.

[61] Tang Y., Guan X. (2009). Parameter estimation for time-delay chaotic system by particle swarm optimization. Chaos Solitons Fractals.

[62] Wang L., Xu Y. (2011). An effective hybrid biogeography-based optimization algorithm for parameter estimation of chaotic systems. Expert Syst. Appl..

[63] Weile D.S., Michielssen E. (1997). Genetic algorithm optimization applied to electromagnetics: A review. IEEE Trans. Antennas Propag..

[64] Kennedy J. (2011). Particle swarm optimization. Encyclopedia of the Sciences of Learning.

[65] Yao X., Liu Y. (1996). Fast Evolutionary Programming. Evolut. Program..

[66] Arı Ç., Aksoy S., Arıkan O. (2012). Maximum likelihood estimation of Gaussian mixture models using stochastic search. Pattern Recognit..

[67] Povinelli R.J., Johnson M.T., Lindgren A.C., Roberts F.M., Ye J. (2006). Statistical models of reconstructed phase spaces for signal classification. IEEE Trans. Signal Process..

[68] Shekofteh Y., Almasganj F. (2013). Feature extraction based on speech attractors in the reconstructed phase space for automatic speech recognition systems. ETRI J..

[69] Shekofteh Y., Almasganj F., Daliri A. (2015). MLP-based isolated phoneme classification using likelihood features extracted from reconstructed phase space. Eng. Appl. Artif. Intell..

[70] Lao S.-K., Shekofteh Y., Jafari S., Sprott J.C. (2014). Cost function based on gaussian mixture model for parameter estimation of a chaotic circuit with a hidden attractor. Int. J. Bifurc. Chaos.

[71] Shekofteh Y., Jafari S., Sprott J.C., Golpayegani S.M.R.H., Almasganj F. (2015). A gaussian mixture model based cost function for parameter estimation of chaotic biological systems. Commun. Nonlinear Sci. Numer. Simul..

[72] Jafari S., Sprott J.C., Pham V.-T., Hashemi Golpayegani S.M.R., Jafari A.H. (2014). A New Cost Function for Parameter Estimation of Chaotic Systems Using Return Maps as Fingerprints. Int. J. Bifurc. Chaos.

[73] Kuznetsov N., Mokaev T., Vasilyev P. (2014). Numerical justification of Leonov conjecture on Lyapunov dimension of Rossler attractor. Commun. Nonlinear Sci. Numer. Simul..

[74] Leonov G., Kuznetsov N., Mokaev T. (2014). Homoclinic orbit and hidden attractor in the Lorenz-like system describing the fluid convection motion in the rotating cavity. arXiv.

[75] Kuznetsov N., Leonov G., Mokaev T. (2015). The Lyapunov dimension and its computation for self-excited and hidden attractors in the Glukhovsky-Dolzhansky fluid convection model. arXiv.

[76] Leonov G., Kuznetsov N., Mokaev T. (2015). The Lyapunov dimension formula of self-excited and hidden attractors in the Glukhovsky-Dolzhansky system. arXiv.

[77] Kuznetsov N. (2016). The Lyapunov dimension and its estimation via the Leonov method. Phys. Lett. A.

[78] Wolf A., Swift J.B., Swinney H.L., Vastano J.A. (1985). Determining Lyapunov exponents from a time series. Phys. D Nonlinear Phenom..

[79] Pincus S.M. (1991). Approximate entropy as a measure of system complexity. Proc. Natl. Acad. Sci. USA.

[80] Pincus S. (1995). Approximate entropy (ApEn) as a complexity measure. Chaos Interdiscip. J. Nonlinear Sci..

[81] Chon K.H., Scully C.G., Lu S. (2009). Approximate entropy for all signals. IEEE Eng. Med. Biol. Mag..

[82] Koyuncu İ., Özcerit A.T. (2016). The design and realization of a new high speed FPGA-based chaotic true random number generator. Comput. Electr. Eng..

[83] Akgul A., Moroz I., Pehlivan I., Vaidyanathan S. (2016). A new four-scroll chaotic attractor and its engineering applications. Opt.-Int. J. Light Electron Opt..

[84] Çavuşoğlu Ü., Akgül A., Kaçar S., Pehlivan İ., Zengin A. (2016). A novel chaos-based encryption algorithm over TCP data packet for secure communication. Secur. Commun. Netw..

[85] Avaroğlu E., Koyuncu İ., Özer A.B., Türk M. (2015). Hybrid pseudo-random number generator for cryptographic systems. Nonlinear Dyn..

[86] Akgul A., Calgan H., Koyuncu I., Pehlivan I., Istanbullu A. (2016). Chaos-based engineering applications with a 3D chaotic system without equilibrium points. Nonlinear Dyn..

[87] Rukhin A., Soto J., Nechvatal J., Barker E., Leigh S., Levenson M., Banks D., Heckert A., Dray J., Vo S. (2010). Statistical Test Suite for Random and Pseudorandom Number Generators for Cryptographic Applications.

[88] Trejo-Guerra R., Tlelo-Cuautle E., Jimenez-Fuentes J., Sánchez-López C., Muñoz-Pacheco J., Espinosa-Flores-Verdad G., Rocha-Pérez J. (2012). Integrated circuit generating 3-and 5-scroll attractors. Commun. Nonlinear Sci. Numer. Simul..

[89] Kantz H., Schreiber T. (2004). Nonlinear Time Series Analysis.

[90] Bishop C.M. (2006). Pattern recognition. Mach. Learn..

[91] Nakagawa S., Wang L., Ohtsuka S. (2012). Speaker identification and verification by combining MFCC and phase information. IEEE Trans. Audio Speech Lang. Process..

[92] Yang X.-S. (2010). Engineering Optimization: An Introduction with Metaheuristic Applications.

[93] De la Fraga L.G., Tlelo-Cuautle E. (2014). Optimizing the maximum Lyapunov exponent and phase space portraits in multi-scroll chaotic oscillators. Nonlinear Dyn..

[94] Mirjalili S., Lewis A. (2016). The whale optimization algorithm. Adv. Eng. Softw..

[95] Mirjalili S., Mirjalili S.M., Hatamlou A. (2016). Multi-verse optimizer: A nature-inspired algorithm for global optimization. Neural Comput. Appl..

[96] Tang W., Wu Q. (2009). Biologically inspired optimization: A review. Trans. Inst. Meas. Control.

